# Rectus Sheath Hematoma: A Rare Surgical Emergency

**DOI:** 10.7759/cureus.12156

**Published:** 2020-12-18

**Authors:** Kyle Drinnon, Sean S Simpson, Yana Puckett, Catherine A Ronaghan, Robyn E Richmond

**Affiliations:** 1 Surgery, Texas Tech University Health Sciences Center, Lubbock, USA; 2 General Surgery, Texas Tech University Health Sciences Center, Lubbock, USA; 3 Surgery, West Virginia University School of Medicine, Charleston, USA

**Keywords:** spontaneous rectus sheath hematoma, abdominal compartment syndrome, surgical case reports, complications of anticoagulation

## Abstract

A rectus sheath hematoma (RSH) is a rare medical condition that consists of blood accumulating in the rectus abdominis muscle sheath. RSH is most frequently due to a hemorrhage from the superior or inferior epigastric artery. RSH has many specific risk factors, such as anticoagulant use. As the use of anticoagulants increases, the incidence of RSH has also increased. This condition can present with the infrequent complication of abdominal compartment syndrome (ACS), which can require surgical decompression of the abdomen to avoid high morbidity and mortality. We present the case of a 79-year-old male who, after receiving anticoagulants, developed a right-sided RSH which progressed to ACS.

The patient was transferred to our care for community-acquired pneumonia, pneumothorax, and increasing respiratory support. He was admitted to the medical intensive care unit (MICU), was placed on a nasal cannula, and given vancomycin and Zosyn for pneumonia. After two days, the patient was switched to enoxaparin for anticoagulation. After three days, the patient's pneumothorax had resolved. At this time, the patient reported swelling in his right lower quadrant (RLQ) with mild pain, nausea, vomiting, and difficulty voiding completely. The physical examination confirmed RLQ swelling, and a kidney, ureter, and bladder (KUB) x-ray and ultrasound were ordered. A CT with and without contrast was also obtained which showed a large right rectus sheath hematoma extension into preperitoneal space and a small amount of intraperitoneal fluid along the right paracolic gutter. Soon after, the patient became lightheaded and fell after using the restroom. Vitals at the time were a blood pressure of 79/56, heart rate (HR) of 127, and oxygen saturation of 88% with his hemoglobin dropping from 11.4 g/dL earlier that morning to 8.4 g/dL. The patient's care was transferred to our surgical team in the surgical intensive care unit (SICU). He received an arterial line, two doses of protamine, 1-liter of crystalloids, and two units of packed red blood cells (PRBC). The patient’s vitals normalized. Interventional radiology (IR) was consulted but they requested the coagulopathy be corrected before any intervention. Reversal of his Lovenox® was thromboelastographic (TEG)-guided and included platelets, cryoprecipitate, and prothrombin complex concentrate/fresh frozen plasma (PCC/FFP), in addition to more PRBCs. During these interventions, the patient acutely decompensated with hypotension, difficulty breathing, and expansion of his hematoma. A bladder pressure in the 30s was obtained, causing him to be sent to the operating room (OR) for decompression, extraperitoneal packing, and the wound was temporarily closed. The patient returned and IR was able to embolize the right inferior epigastric artery. The patient was taken to the OR again for exploration, removal of packing, and closure.

RSH is a rare complication that can occur due to trauma, coagulopathy, obesity, and muscle strains during a pregnancy. Larger hematomas tend to occur below the arcuate line because there is an absence of the posterior rectus sheath which enables the hematomas to spread. An RSH can be treated with conservative measures, but for patients who continue to bleed, more aggressive measures should be taken to avoid life-threatening complications, such as ACS.

## Introduction

Rectus sheath hematomas (RSH) are an uncommon pathology [1-3]. The presentation of RSH varies but the patients frequently have one or more of the following risk factors: trauma, coagulopathy disorder, obesity, cough, or pregnancy. Patients are usually managed conservatively, but there are rare cases in which surgical intervention is required. Herein, we present a case of RSH with the rare complication of abdominal compartment syndrome (ACS), requiring both surgical and endovascular intervention. In over six million patients with conditions such as atrial fibrillation, mechanical heart valve, or venous thromboembolism, anticoagulation is a key component in their therapy. With the increased use of anticoagulation, there has been an increase in the incidence of RSH [4]. Most uncomplicated cases can be managed conservatively. The increased incidence of RSH should also increase the number of complications associated with them. ACS is a relatively rare complication, but it also represents a significantly morbid complication.

## Case presentation

A 79-year-old male was transferred to our hospital for management of his community-acquired pneumonia, pneumothorax, and increasing respiratory distress. The patient had a past medical history of hypertension, hyperlipidemia, and atrial fibrillation for which he was taking Coumadin blood thinner and had a pacemaker. He had initially presented to an outside facility with a two-week history of high fever, shortness of breath, cough with bloody sputum, and lack of appetite with no signs of headache, abdominal pain, nausea, vomiting, or weight loss.

**Figure 1:**
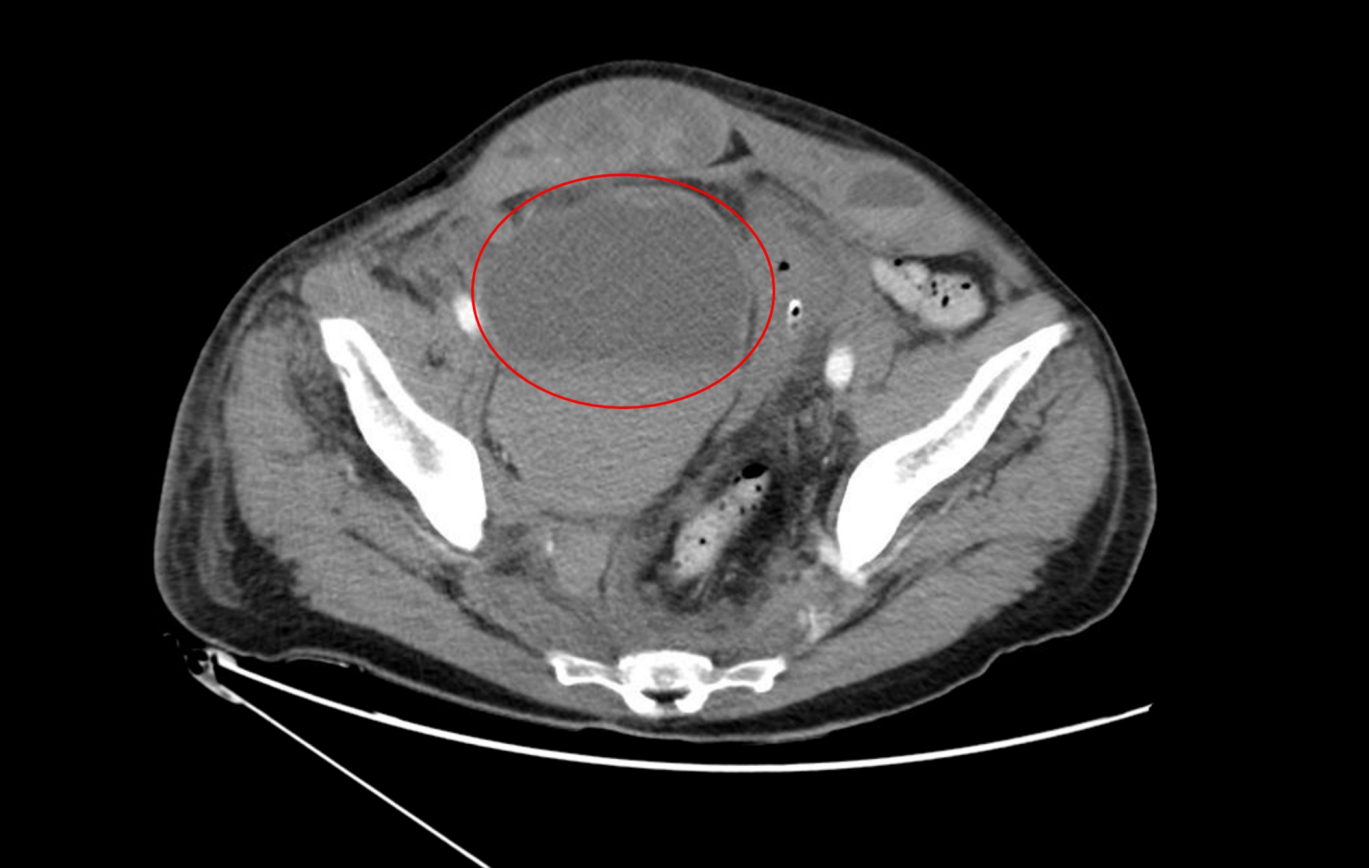
Contrast-enhanced CT pelvis (axial) showing right-sided rectus sheath hematoma (red circle) with extension into the right paracolic gutter.

He was admitted to our medical intensive care unit (MICU), and placed on a high flow nasal cannula (NC). Since his pneumonia had not initially responded to management at the outside facility, he was placed on broad-spectrum antibiotics. On Day 2 of his hospitalization, the patient was stable but still had a cough and leukocytosis. He was switched to enoxaparin for anticoagulation. By Day 3, he was liberated from high flow, his pneumothorax had resolved, and his leukocytosis was improving. The patient did notice some swelling in his right lower quadrant associated with mild pain. On Day 4, he reported the swelling and discomfort to the MICU team. He also later reported nausea and emesis. He had trouble with urination. Physical examination was significant for right lower quadrant (RLQ) swelling with a rough area of 10 x 10 cm, with no overlying skin changes or significant tenderness. The workup for the mass began with a kidney, ureter, and bladder (KUB) x-ray and an abdominal wall ultrasound. Ultrasound revealed a complex right lower quadrant structure measuring 15.1 x 11.5 x 4.4 cm with internal Doppler flow, a computerized tomography scan (CT) of the abdomen and pelvis with and without contrast was obtained and our surgical team was consulted. The CT showed a large right rectus sheath hematoma extension into preperitoneal space and a small amount of intraperitoneal fluid along the right paracolic gutter (Figure 1). 

**Figure 2:**
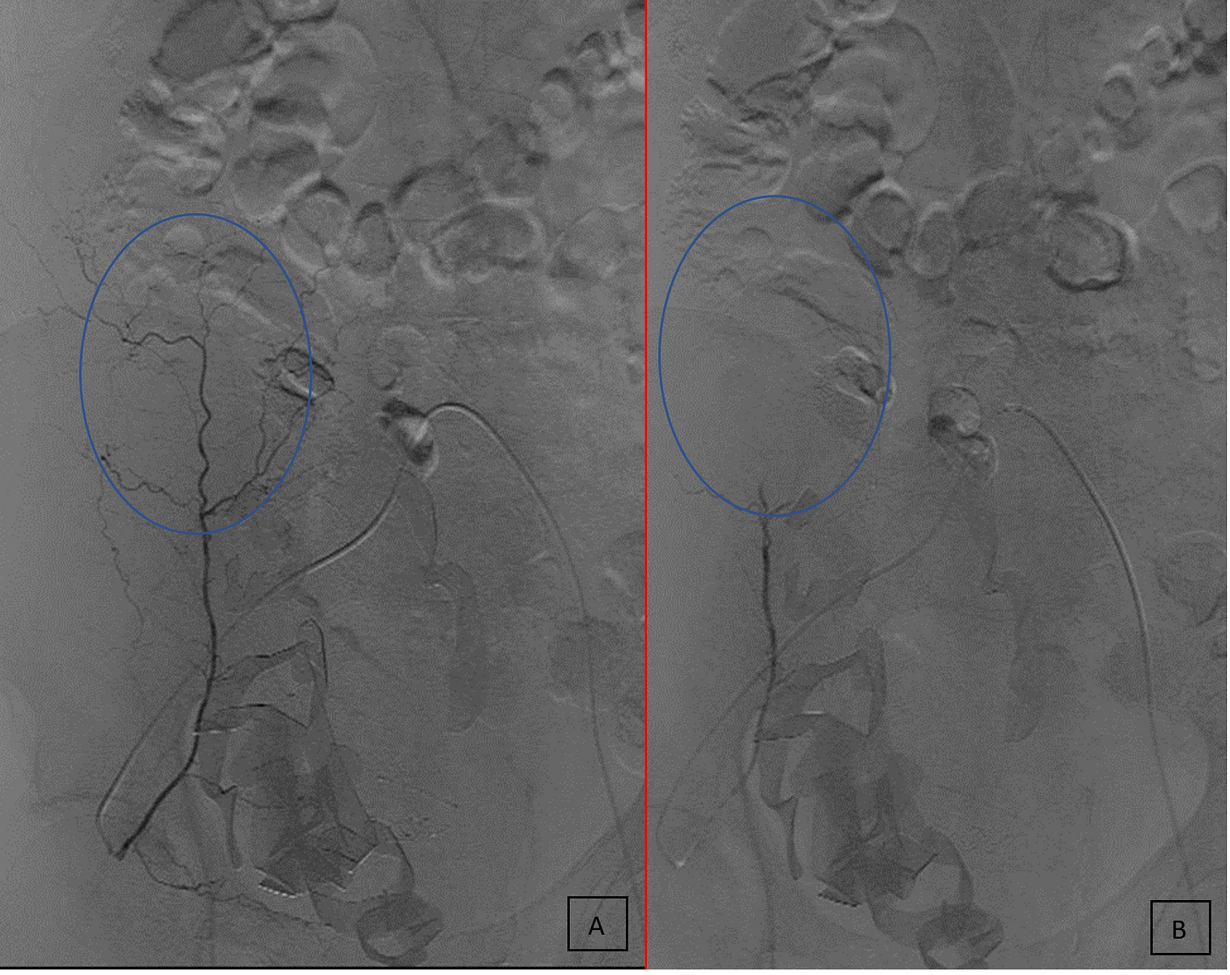
Angiography showing isolation of the right inferior epigastric artery before (A) and after (B) embolization.

After his CT scan, the patient had an orthostatic event, where he felt lightheaded and fell after using the restroom. He was obtunded and pale when he was initially evaluated. His vital signs revealed a blood pressure of 79/56, heart rate of 127, and oxygen saturation of 88%. He was placed on 5 liters (L) of oxygen delivered through a nasal canula, a fluid bolus was initiated, and laboratory studies were drawn. His hemoglobin was found to be 8.4 g/dL, down from 11.4 g/dL earlier that morning due to blood loss later found to be due to a hematoma. At this point, the patient’s primary care was transferred over to our surgical team, and he was transported to our surgical intensive care unit (SICU) where we could continue his resuscitation and place an arterial line. He then received two doses of protamine for anticoagulation reversal, 1 L of crystalloid, and two units of packed red blood cells (PRBC). His vitals normalized, and he became more alert. Interventional radiology (IR) was consulted. Reversal of his anticoagulation was thromboelastographic (TEG)-guided and included platelets, cryoprecipitate, and prothrombin complex concentrate (PCC). Throughout that day, the patient had oliguria; early into the next morning, he developed anuria. Several hours after his initial resuscitation, the patient acutely decompensated with hypotension, difficulty breathing, and expansion of his hematoma. Bladder pressure was obtained, which was in the 30s, so the decision was made to take him to the operating room (OR) for decompression. The patient was explored and the hematoma evacuated. The abdomen was packed with laparotomy pads and the abdomen was left open temporarily with a wound vacuum in place. Upon return from the OR, his resuscitation was continued until he was taken to interventional radiology. His right inferior epigastric artery was embolized (Figure 2). Later that evening, he was taken back to OR for exploration, removal of packing, and closure. The patient required additional postoperative critical care but ultimately recovered and was sent home with home health care.

## Discussion

RSHs are an uncommon clinical event, but they are an important pathology to consider when evaluating a patient with abdominal pain. A risk factor that should raise clinical suspicion is muscle strain, such as a strain due to chronic cough, as seen in our patient. Other risk factors include involvement in trauma, coagulopathy, obesity, and pregnancy [1]. Most of these risk factors all have the potential to introduce significant shear stress on the superior or inferior epigastric arteries and their associated branches anastomosing between the muscle and posterior layer of the rectus sheath or direct injury to the muscle itself [2, 5-6]. Coagulopathic patients typically have one of the aforementioned risk factors, but their natural ability to stop the bleeding is inhibited.

RSHs above the arcuate line are generally a result of damage to the superior epigastric artery. Below the arcuate line, the inferior epigastric vessels tend to be the source of bleeding. Large hematomas tend to occur below the arcuate line due to the absence of a posterior rectus sheath, enabling hematomas to spread extraperitoneally or even intraperitoneally should the peritoneum be ruptured [7-8]. Hematomas below the arcuate line can dissect into the retroperitoneal space where large quantities of blood may be lost before outward evidence of a hematoma is detectable. Hemorrhage into this area also may minimize any natural tamponade effect.

The main features of RSH are abdominal pain with a mass; beyond that, the symptoms can be quite variable. Signs that can be seen in our patient and other patients with RSH and ACS include some of the following: fever, chills, weakness, confusion, pallor, diaphoresis, and abdominal pain. Abdominal bruising is a late sign. It may be periumbilical in Cullen’s sign or the flanks in Grey-Turner sign, indicating intraperitoneal rupture of an extraperitoneal extension. If clinical suspicion is high, appropriate imaging studies include abdominal ultrasound and CT scan. Treatment is usually conservative, including rest, analgesia, fluid resuscitation, transfusion if necessary, and correction of any coagulopathy. Carnett’s test can help distinguish if the pain is a result of intraperitoneal or arising from the abdominal wall. Fothergill’s sign is helpful in distinguishing between an intraabdominal mass and a mass from the rectus sheath [4].

## Conclusions

Based on the case we presented, conservative measures may not be sufficient in the treatment of ACS due to RSH. The use of surgery or other aggressive measures should be evaluated quickly if conservative measures prove ineffective since serious complications can arise. Our patient initially responded to resuscitative measures but then developed the rare complication of ACS. Continuing conservative measures would have led to poor outcomes for our patient, and based on these findings, future patients suffering from similar medical issues should be considered for surgery or other aggressive treatment options. 
